# Impact of Using the Intelligent Physical Health Measurement System on Active Aging: A Survey in Taiwan

**DOI:** 10.3390/healthcare9091142

**Published:** 2021-09-01

**Authors:** Wen-Chou Chi, Wei-Chen Cheng, Ting-Hung Chen, Po-Jin Lin

**Affiliations:** 1 Department of Occupation Therapy, Chung Shan Medical University, Taichung 40201, Taiwan; dannychi@csmu.edu.tw; 2 Occupational Therapy Room, Chung Shan Medical University Hospital, Taichung 40201, Taiwan; 3 Department of Information Management, National Chung Cheng University, Chiayi 62102, Taiwan; zwc0316@gmail.com; 4 Institute of Health Policy and Management, College of Public Health, National Taiwan University, Taipei 100, Taiwan; quwar86489@gmail.com; 5 Department of Sports, Health and Leisure, Tatung Institute of Technology, Chiayi 600, Taiwan

**Keywords:** long-term care 2.0, intelligent physical health measurement system, active aging

## Abstract

Background: In Taiwan, the Chiayi City Government and Industrial Development Bureau of the Ministry of Economic Affairs have worked together to promote smart health management in the community and encourage people to use the intelligent physical health measurement system (IPHMS) with Smart Body Health Measuring Machine. Volunteers help participants in the community to use the IPHMS to ensure that measurements are taken correctly. Objectives: This study aimed to explore volunteers’ satisfaction with using the IPHMS and the effects of the measurement service on the participants’ measurement behavior intention, and further explore the impact on their active aging. Methods: This study used a paper questionnaire to survey both the participants of the measurement service and the community volunteers from March to April 2021. A total of 180 valid responses were collected. Results: The sociodemographic information showed that the volunteers were mostly female, were aged over 61 years old, had received junior college education, had spent less than 3–6 years in community service, and had 6 months to 1 year of measured service experience. Additionally, the participants of the measurement service were mostly female, were aged over 61 years old, had received below middle school education, had spent less than 1–3 years in community service, and spent an average of 5 days in the community each week. Our results showed that the information quality (β = 0.352, *p* < 0.001) and system quality (β = 0.701, *p* < 0.001) had significant effects on volunteers’ satisfaction of using the IPHMS. Subjective norms had significant effects on participants’ perceived disease threat (β = 0.347, *p* < 0.001) and behavior intention of management service (β = 0.701, *p* < 0.001); furthermore, behavior intention had significant effects on their social participation for active aging (β = 0.430, *p* < 0.05). Conclusions: Improving the system and information quality is likely to improve volunteers’ satisfaction with the system. Active aging factors only affect social participation, which represents the measurement services promote for social interaction mostly.

## 1. Introduction

In 2016, Taiwan implemented the Long-Term Care Policy 2.0 to expand the system’s original service targets, increase service hours, develop innovative services, and achieve multiple continuous services centered on service users [1]. In addition to service targets mostly focused on the elderly, the Taiwan government considered the needs of special groups and targeted the entire possible population with service needs, such as people with dementia aged over 50, plain-land indigenous people with functional limitations (aged 55 to 64), people with disability (aged under 49), and older people with frailty (aged 65 and over) [2,3]. The Community Overall Care ABC Model for long-term care policy 2.0 expanded these services to every corner and established an integrated service center in the community (A-level long-term care is the flagship unit) through the participation of the community, the expansion of a compound service center (B-level long-term care is a specialty unit), and the implementation of many long-term care stations (C-level long-term care is a convenience unit) in small alleys and lanes, as shown in Table 1 [4]. Among them, C-level care units are intended for long-term care in policy 2.0. C-level care units are located within neighborhoods. The services provided include convenient care, temporary respite care, extended preventive disability-delaying activities, and communal dining or meals on wheels for households [5]. C-level care units can help people in the community maintain and enhance their personal self-care ability, improve their self-efficacy concerning health, and better understand the degree of self-managed active aging in the community. The quality of life of caregivers is constantly being considered and improved in the overall community care model for more actively deploying a dense care network [6].

In order to enhance the improved “Long-Term Care 2.0” plan, Chiayi City Government and Industrial Development Bureau of the Ministry of Economic Affairs worked together to implement smart care services in Chiayi City [7]. They developed an intelligent physical health measurement system (IPHMS) with Smart Body Health Measuring Machine in long-term care C-level stations to provide health measurement services that could be used at any time and place. Volunteers in the community were service providers; they help qualified citizens to use the IPHMS. Therefore, this study mainly explored this topic from two angles: We explored the volunteers’ satisfaction with the IPHMS based on the information system success model and adopted perceived disease threat in the Health Belief Model and subjective norms in the Theory of Planned Behavior to explore participants’ behavior intention of the measurement service and further explored the impact on their active aging.

## 2. Theoretical Framework and Hypothesis Development

### 2.1. Information System Success Model (ISSM)

In recent years, the information system success model (ISSM) created by DeLone and McLean [8] has been used in medical information departments and the explanatory power and fit of the models have been quite high [9]. Wixom and Todd [10] indicated that system quality included the dimensions of reliability, flexibility, convenience, ease of use, and functionality. DeLone and McLean [11] indicated that the dimensions of information quality included informativeness, readability, clarity, content, conciseness, completeness, and precision. According to Hsieh et al. [12], in a study on a successful model of a community telemedicine system, the quality of the information and system affected user satisfaction. Kurt [13] found that information quality and system quality had a significant impact on user satisfaction. It was also found that user satisfaction and system usage had a positive and significant impact on the success of the system.

Although many studies have used ISSM to explore the success of such systems, Yeh et al. [14] pointed out that in the context of medical information technology the use of SERVQUAL for measuring service quality is still controversial and must be discussed in depth in three dimensions: interactive quality, physical environment quality, and result quality. Lian [15] found that the alignment between usefulness and service quality had no significant impact on nurses’ satisfaction because the hospital required a fixed use relationship, meaning that the quality of service did not affect satisfaction. Since there is no interaction between users and system personnel, this study only explored the impacts of the information quality and system quality on user satisfaction. The related hypotheses were proposed as follows:

**Hypothesis** **1a** **(H1a).** *The “information quality” of IPHMS positively affects volunteers’ “user satisfaction”*.

**Hypothesis** **1b** **(H1b).** *The “system quality” of IPHMS positively affects volunteers’ “user satisfaction”*.

### 2.2. Health Belief Model (HBM) and Theory of Planned Behavior (TPB)

HBM and TPB are widely used in the field of health psychology and have gradually been applied to information management [16,17,18]. Much prior research used HBM in combination with TPB in the study of health behavior to understand users’ beliefs, values, and attitudes regarding a wide range of health-related behaviors [19]. Santi Lozoya et al. [12] studied the impact of smartphone apps on the oral health behaviors of parents of preschool children and found that subjective norms positively affect parents’ behavioral intentions. Additionally, Zhao and Zhou [20] studied mobile medical services for the elderly and found that the perception of usefulness, ease of use, subjective norms, trust, perceived threats, and attitudes seriously affect behavioral intentions. Wang et al. [18] found that HBM affects people’s perception of the threat of disease, which, in turn, affects the possibility of patients taking recommended health prevention behaviors, while perception of disease threat also affects people’s intent to use remote health monitoring services. Based on these prior studies, this study mainly focused on perceived disease threat, subjective norms, and behavioral intentions in the HBM and TPB. Therefore, the related hypotheses were proposed as follows:

**Hypothesis** **2a** **(H2a).** *The “subjective norms” of participants in long-term care 2.0 level-C stations positively affect their “perceived disease threat”*.

**Hypothesis** **2b** **(H2b).** *The “subjective norms” of participants in long-term care 2.0 level-C stations positively affect their “behavioral intentions” to use the IPHMS.*

**Hypothesis** **3** **(H3).** *The “perceived disease threat” of participants in long-term care 2.0 level-C stations positively impacts their “behavioral intention”.*

### 2.3. Active Aging

Marsillas et al. [21] indicated that active aging involved (1) reducing the incidence of disease and disability; (2) maintaining good mental and physical functions; and (3) having an active life and social participation. In view of the development trend of global population aging, the World Health Organization [22] published ‘’Active Aging: A Policy Framework’’, which included the three pillars of active aging—health, participation, and safety—and proposed the six determinants of active aging: (1) health and social services (such as health promotion, disease prevention, curative services, long-term care, and mental health services); (2) the behavioral dimension (such as smoking and tobacco usage, physical activity, healthy eating, oral health, alcohol, and medication); (3) the personal dimension (such as biology, genetics, and psychological factors); (4) physical environment (friendly environment, safe housing, falls, and the absence of pollution); (5) the social dimension (e.g., social support, violence and abuse, and education and literacy); (6) the economic dimension (e.g., income, social protection, elder workforce employment).

Over the last few decades, active aging has been explored further, and it has played an increasingly important role not only in research, but also in policy and society. This contributes to the life satisfaction of the elderly. In this study, we tested new models of active aging. Active aging is often regarded as an advanced concept of successful aging, covering the three aspects of health, psychology, and society. However, the concept of active aging considers the nature of interaction with the living environment, including opportunities for elderly citizens to feel a sense of value in society [23]. The new model proposed by Marsillas et al. [21] includes the aspects of healthy life, cognitive style, emotional state, social participation, lifelong learning, use of technology, employment, and leisure activities. From these aspects, it can be seen that health, life, social participation, physiology, and psychology are the most commonly used aspects for assessing active aging in both old and new research.

Petretto et al. [24] indicated in their development model of active aging for the elderly that the main feature that should be considered is the concept of psychological function, including the meaning of life, psychological feelings, and negative treatment, which all have an impact on active aging. Chuang and Chen [25] believe that good family relationships and interpersonal interactions are important aspects of social participation. They believe that people must continue to participate in different activities to achieve the goals of active aging through labor participation, social participation, and even life participation [21]. The meaning of health includes adopting a healthy lifestyle and taking part in active health management. According to the positive definition of health proposed by scholars, active aging not only reduces the occurrence of disease and disability but also helps to maintain good physical and mental functions to further promote social development [26]. Zaidi and Howse [27] emphasize that successful aging depends on individuals having sufficient material and non-material resources, and this sense of security comes from the state of government support. The impacts of economic conditions on active aging are significant.

In the past few years, the use of Information and Communication Technology (ICT) has become very important, as it enables older people to stay connected to society and to their social networks [28]. Gjevjon et al. [29] considered the use of ICT as a predictor of active aging. Chen and Tang [30] used active aging to evaluate system requirements based on the construction of a mobile application system for health and found that the system assisted active aging. These studies showed that active aging can be improved by information systems. Therefore, we sorted out the relevant aspects of active aging. The five aspects were psychology, life, social participation, health, and economics. These aspects can be used to understand the influence of IPHMS on active aging. Therefore, the related hypotheses were proposed as follows:

**Hypothesis** **4a** **(H4a).** *Long-term care 2.0 level-C station participants’ “behavioral intentions” of using the IPHMS positively affects their “active aging: psychology” aspect.*

**Hypothesis** **4b** **(H4b).** *Long-term care 2.0 level-C station participants’ “behavioral intentions” of using IPHMS positively affects their “active aging: life” aspect.*

**Hypothesis** **4c** **(H4c).** *Long-term care 2.0 level-C station participants’ “behavioral intentions” of using the IPHMS positively affects their “active aging: social participation” aspect.*

**Hypothesis** **4d** **(H4d).** *Long-term care 2.0 level-C station participants’ “behavioral intentions” of using IPHMS positively affects their “active aging: health” aspect.*

**Hypothesis** **4e** **(H4e).** *Long-term care 2.0 level-C station participants’ “behavioral intentions” of using IPHMS positively affects their “active aging: economy” aspect.*

**Figure 1:**
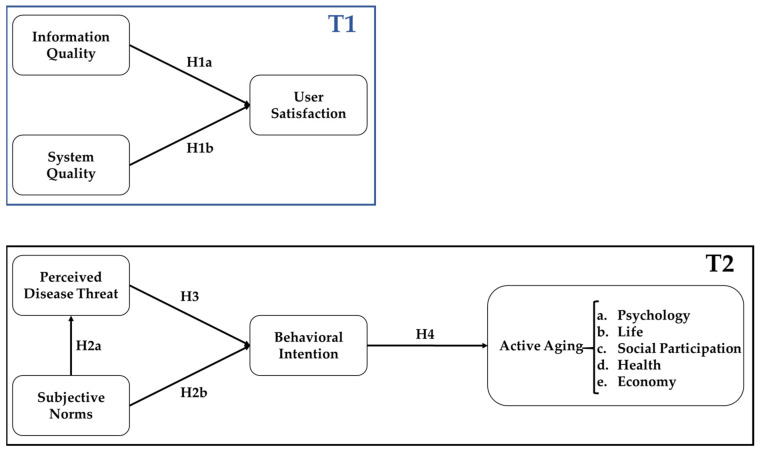
Research framework.

Volunteers in the community are service providers; they help the participants to use the IPHMS. Thus, our research framework can be divided into two parts (Figure 1): the first part (T1 model) is based on the use of the ISSM to evaluate community volunteers’ satisfaction with the IPHMS; the second part (T2 model) is based on the HBM and active aging to measure participants’ behavior intentions and active aging who use the IPHMS.

## 3. Research Methodology

### 3.1. Introduction of Case Studies

**Figure 2:**
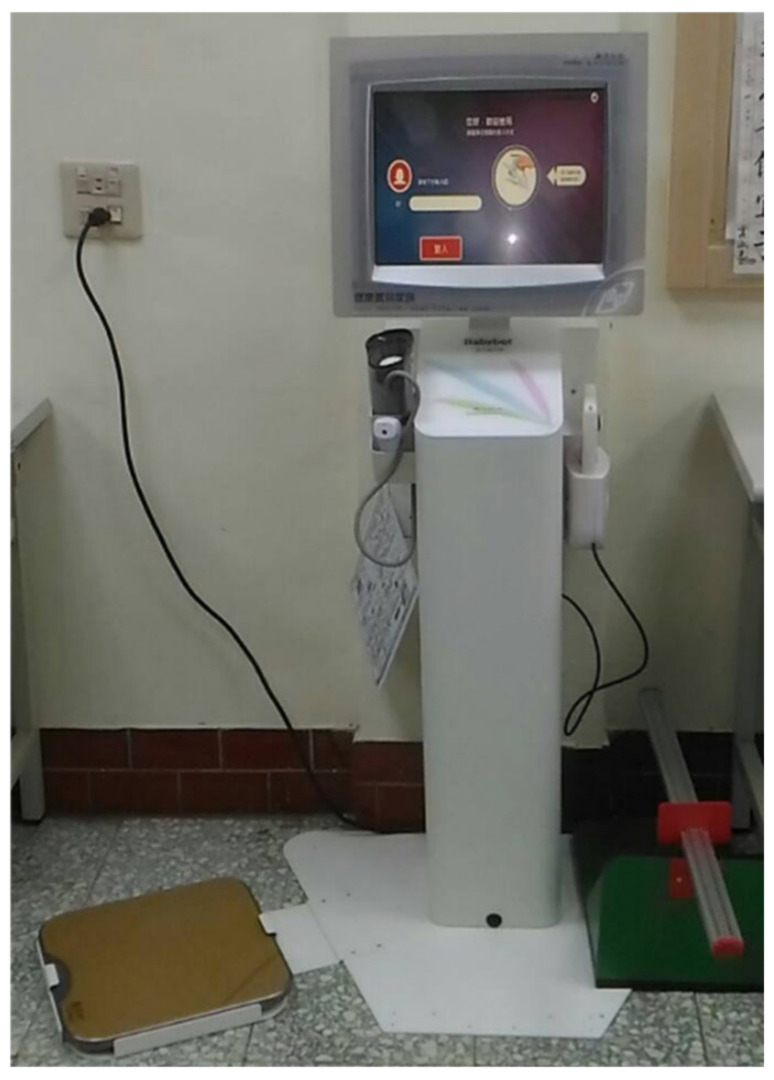
Smart Body Health Measuring Machine.

Taiwan’s Ministry of Health and Welfare proclaim that the number of C-level care stations is expected to be 2,529 [1]. This will reduce the burden on caregivers and be useful for the elderly. Long-term care is carried out for the purpose of “safe aging in place”. Therefore, the IPHMS is important for assisting community care. This system contains “Smart Body Health Measuring Machine” in this plan (see Figure 2). Participants need to log in with their National Health Insurance Card. The measurement data are transmitted to the health measurement database through a wireless network. When the participant wants to measure health indicators in the future, there are two ways to log in: one is to insert their health insurance card, the other is to enter the ID number on the screen and the device. The system can measure seven items: blood pressure, grip strength, forehead temperature, sitting and standing speed, walking speed, body flexibility, and body fat. Volunteers mainly assist the participants operating the system interface and key in the measurement data. In particular, the process of measuring body flexibility requires two people to complete the task, with the participant bending and the volunteer measuring and keying in the data. After all measurements are completed, the participant can preview the results and view the latest seven records.

In Chiayi City, a total of 30 locations have been set up under this project. The bases include development associations, care stations, government units, medical centers, and hospitals. As this research is “people-centered” and is a service object recognized by C-level stations, there are 15 bases selected in total in this research.

### 3.2. Subjects

The subjects can be divided into two groups: The first group consisted of volunteers in the community to operate the IPHMS and the inclusion criterion was that they must have a volunteer training certificate. The second group consisted of participants of the measurement service and the inclusion criterion was that they must be qualified for long-term care 2.0 policy. Firstly, this research sorted out the locations where the sub-project of the “Silver Hair Sharing Life Circle Service Project” of the “Home Measurement and Evaluation of Medical Diseases-New Smart Health Service Platform Project” was implemented. As aforementioned, sustainable local health care and care services based on a “people-centered” approach were selected, as well as community care centers of the “silver-haired life care circle” centered on the base [7]. We then confirmed the participants of the measurement service and volunteers in the area used IPHMS with the assistance of the community director or director general, and sent out a questionnaire to them after obtaining their consent.

### 3.3. Questionnaire Design

Two versions of questionnaire were developed based on questionnaire items with good reliability and validity in past research. Additionally, according to the goal of this research and the direction we explored, the interpretation and modification of the questionnaire items were carried out. After the first draft of the questionnaire was completed, we invited experts in health information, the management information system, and long-term care to review the content of our questionnaire for any ambiguities and or inappropriateness.

The first questionnaire was used to survey the volunteers in the community; it consisted of 10 model-related items (see Appendix A Table A1) and an additional five items on demographic information such as age, gender, education level, years of community service (participation), and measured service experience. The second questionnaire was used to survey the participants of the measurement service who used the equipment; it consisted of 28 model-related items (see Appendix A Table A2) and an additional five items on demographic information such as age, gender, education level, years of community service (participation), average number of days in the community a week, and using body measurement equipment frequency. Each item was measured on a 5-point Likert scale (from 5, strongly agree, to 1, strongly disagree).

### 3.4. Data Collection and Analysis

In this study, we distributed a paper questionnaire from March to April in 2021. Relevant personnel and volunteers who did not participate in this study assisted in reading the content to some elderly participants. Data were processed using SPSS 22.0 [31] for descriptive statistical analysis. SmartPLS 3.2.6 [32] was used for reliability/validity analysis and structural equation modeling analysis, including confirmatory factor analysis, confirmatory causal modeling, and hypothesis testing.

In addition, in order to better understand the results and psychology of the participants, we conducted an informal follow-up interview of the active participants to provide more specific suggestions after the questionnaire. We interviewed three community volunteers. The interview contained only one question relating to how they felt about the project.

## 4. Results

As aforementioned, different questionnaire was sent to two groups: volunteers and participants of the measurement service. The number of valid responses to volunteer and participant were 55 (the valid recovery rate was 84.6%) and 125 (the valid recovery rate was 83.3%), respectively. This study analyzed the results of the two sets of valid questionnaires after confirmation.

### 4.1. Descriptive Statistical

Volunteers were mostly female (70.9%), aged over 61 years (58.2%), with a junior college education (40.0%), with less than 3–6 years of community service (participation) (41.8%), and a total of 6 months to 1 year of measured service experience (49.1%). Participants of the measurement service were mostly female (70.9%), aged over 61 years (85.6%), with below middle school education (62.4%), less than 1–3 years of community service (participation) (39.2%), and spent an average number of more than 5 days in the community each week. Table 2 shows the results of the descriptive statistical analyses of the two samples.

### 4.2. Reliability and Validity Analysis

We conducted a reliability and validity analysis using SmartPLS 3.2.6. We found a few items with factor loadings less than 0.7, so we removed these items according to the suggestion of Hair et al. [33]. After removing the items (AA5), the results showed that the related factor loadings were >0.7, the composite reliability (CR) values were >0.7, and Cronbach’s α was above 0.7 [33]. Although Cronbach’s α for subjective norms was 0.634, this was also considered to be medium reliability and was still acceptable. The average variance extracted (AVE) of each dimension was >0.5 [33] and the square root of AVE was greater than the relevant coefficients of other dimensions [34]. Therefore, our questionnaire had a reasonable reliability and internal consistency and an acceptable discriminability and convergent validity, as shown in Table 3 and Table 4.

**Remark** **1.**

*1.*  *The diagonal line in bold is the square root of the average variance extracted (AVE) value of the facet [34].*
*2.*  *The value under the diagonal is the correlation coefficient of the factor facet.*
*3.*  *The comparison of each aspect and the code abbreviation is as follows: IQ: information products; SQ: system quality; US: user satisfaction; BI: behavioral intention; PDT: perceived disease threat; SN: subjective norms; AA-Phy: active aging psychology; AA-life: active aging life; AA-social: active aging-social participation; AA-Health: active aging health; AA-econ: active aging economy.*

### 4.3. Structural Model Analysis

This study used SmartPLS 3.2.6 to conduct the Partial Least Squares (PLS) analysis of paths between the variables and determined interrelationships among factors and the explanatory power of the model. According to the suggestion of Hair et al. [33], the bootstrap repeated sampling method was carried out 5000 times to estimate the parameters and test the hypotheses.

**Figure 3:**
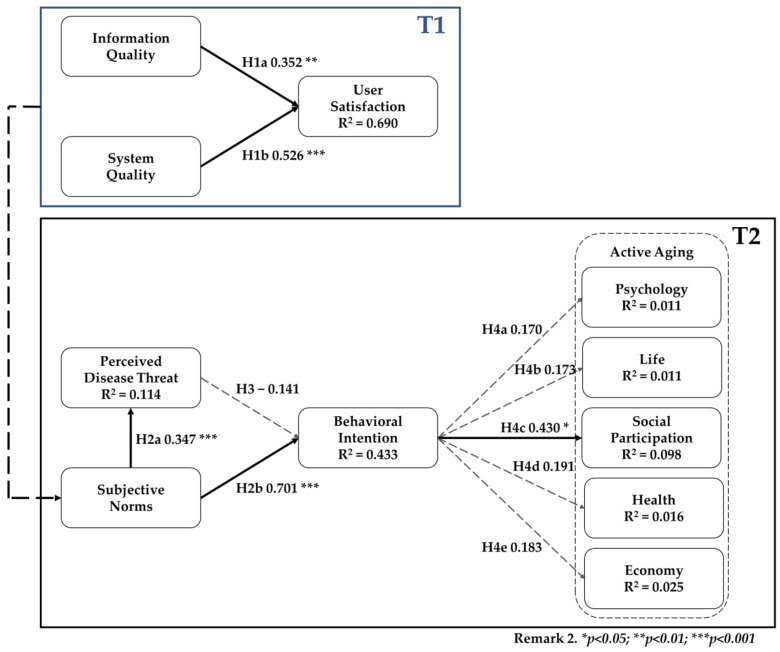
Results of the model.

In the T1 model, we found information quality (0.352; *p*-value = 0.004) and system quality (0.526; *p*-value = 0.000) to have positive significant effects on user satisfaction, meaning that H1a and H1b were accepted. The T1 model was able to explain 69.0% of the total variance of user satisfaction. The results are presented in Figure 3 and Table 5.

In the T2 model, we found that subjective norms had a significant positive effect on perceived disease threat (0.347; *p*-value = 0.000) and behavior intention (0.701; *p*-value = 0.000), meaning that H2a and H2b were accepted. However, perceived disease threat had no effect on behavior intention. Furthermore, behavior intention had no effect on the four aspects of active aging, including psychology, life, health, and the economy, but it had a positive effect on active aging for social participation (0.430; *p*-value = 0.036), meaning that H4c was accepted. The T2 model was able to explain 9.8% of the total variance of active aging for social participation. The results are presented in Figure 3 and Table 5.

**Table 5:** Path analysis results list.

Hypothesis		Path Coefficient	*T*-Value	*p*-Value	R Square
H1a	IQ → US	0.352	2.868	0.004 **	0.690
H1b	SQ → US	0.526	3.941	0.000 ***	
H2a	SN → PDT	0.347	4.459	0.000 ***	0.114
H2b	SN → BI	0.701	6.112	0.000 ***	0.433
H3	PDT → BI	−0.141	1.247	0.212	
H4a	BI → AA-Phy	0.170	0.966	0.334	0.011
H4b	BI → AA-Life	0.173	0.963	0.335	0.011
H4c	BI → AA-Social	0.430	2.099	0.036 *	0.098
H4d	BI → AA-Health	0.191	1.012	0.312	0.016
H4e	BI → AA-Econ	0.183	0.992	0.321	0.025

**Remark** **2.**

*1.*  * p < 0.05; ** p < 0.01; *** p < 0.001
*2.*  *The comparison of each aspect and the code of abbreviations are as follows: user satisfaction (US); behavioral intention (BI); perceived disease threat (PDT); active aging psychology (AA-Phy); active aging life (AA-Life); active aging social participation (AA-Social); active aging health (AA-Health); active aging economy (AA-Econ).*

### 4.4. Informal Follow-Up Interview for Subjects

We then interviewed three community volunteers. The basic information of the interviewees is shown in Table 6.

The interview results showed that there were more than 20 regular users in each community using the measurement services, and they all thought that the “complexity is too high.” However, this part concerned the feelings of the elderly in the community, not the perspectives of the volunteers. The elderly participants recognized that most people do not understand the equipment and are impatient with the queues to use the equipment, but would use the equipment with the volunteers’ encouragement and assistance. They suggested that rules should be enforced before implementing each project and that this equipment in the community should be made use of. This is a major factor in the participants’ subjective norms (See Figure 3).

Meanwhile, participants hope that the system can integrate their data and provide benefits of participating in the project for them and their family members. Although paper-based copies were provided, the recommendations did not come in time for the family members to participate. The aim of “combining the system with life” is very important. Most of the participants appreciated the social interaction brought by the project and felt a very deep feeling of warmth between people, which was seldom found in the community.

## 5. Discussion and Conclusions

### 5.1. Discussion

The results of this research show that both information quality and system quality have a positive impact on user satisfaction. The volunteers representing the community had positive perceptions of using the IPHMS, and their satisfaction was high. This result is in compliance with Lian’s finding [15] that the information quality and system quality have a direct impact on user satisfaction.

Additionally, the result of HBM shows that the participants in the community will be influenced by other people they consider important. People around them pay special attention to their health or assist them in using equipment. Therefore, subjective norms have a positive impact on the elderly. In addition, participants in activities believe that their health is not threatened. They did not have negative thoughts and feelings about their own health, only when they were affected by others. Therefore, the threat of perceived diseases is low. We found with regard to the influence of active aging factors that only the elderly had a significant impact on social participation. The elderly who were willing to use the measuring services had increased social interaction with volunteers or other people. On the contrary, if the elderly had a low willingness to use the equipment, they felt disgusted.

The results of the informal interview carried out afterward showed that the behavioral intentions of the participants of the measurement service to use the equipment was influenced by subjective norms. The interview revealed that the assistance or suggestions of volunteers may influence these subjective norms. Therefore, T1 may affect T2, which was explored by the later interviews. Meanwhile, the survey results showed that active aging factors affect only social participation and not other aspects. In the follow-up review, most participants reported that they were healthy and had a sufficient economic status. They reported that they liked to participate in leisure and entertainment activities rather than curriculum activities or common-sense promotion. This may help to explain H4: Participants’ intention to use the equipment only affected the social participation category in terms of the active aging factors and not others.

### 5.2. Conclusions

This study indicated that the system quality and information quality were useful for explaining the factors affecting volunteers’ satisfaction with the IPHMS, while their assistance or suggestions may influence participants’ subjective norms. participants’ subject norms impacted their perceived disease threat and behavioral intentions, and behavioral intentions also impacted social participation with active aging. Since the global COVID-19 outbreak has occurred, the community epidemic prevention is one of the most important aspects of health and safety. The IPHMS in the community can help qualified citizens achieve self-health management. In particular, the health measurement system can aid with the epidemic prevention strategy formulated by the central government during the COVID-19 epidemic. Elderly can join in community epidemic prevention efforts through autonomous health measurement, further establishing an epidemic prevention network between people in the community.

## 6. Limitation and Direction of Future Studies

Although the participants were all able to be subsidized by Taiwan’s long-term care policy 2.0, in future studies more research is needed for the age group of 65 and above to further differentiate the detailed needs of different age groups. Since this age group is the target group of the long-term care policy 2.0, differentiating the detailed needs of different age groups can serve as a reference for policy makers. In addition, many of the subjects recruited might be in favor of the study or desire social interactions. The potential bias of this study is that we had no way to access the opinions of those who did not participate in this study about the project. Therefore, precautions should be taken when generalizing the results of this study. 

This study mainly used a structured questionnaire to collect data, so it may not have been possible to measure people’s overall substantive feelings about objects in all communities. DeLone and McLean [8] indicated that service quality is an effective service by which information system personnel can provide services to users. The volunteers in this study were servicer workers, and we had no way to access their opinion about the service quality of the system. Therefore, we recommend that future studies should expand the scope of the model’s inference, such as information system personnel and government units.

## Figures and Tables

**Table 1:** Setting and service provided for each level of long-term care unit.

Long-Term Care Unit	Setting	Service
A-level	Hospital Rizhao center Health center	Form a community health care team Provide B and C medical resources
B-level	Day care center for the elderly Nursing home Rehabilitation center Clinic	Provide day service Relief services for the disabled, provide meals, physical rehabilitation, etc.
C-level	Home care station Community care base	Provide short-term care and care Community health promotion, caring visits, catering services, entertainment, and leisure, etc.

Source: Ministry of Health and Welfare [4].

**Table 2:** Descriptive statistical analysis.

	Volunteers (N = 55)		Participants of Measurement Service (N = 125)	
	n	%	n	%
Age				
Under 30 years old (inclusive)	1	1.8%	0	0.0%
31–40 years old	5	9.1%	1	0.8%
41–50 years old	8	14.5%	1	0.8%
51–60 years old	9	16.4%	16	12.8%
Over 61 years old	32	58.2%	107	85.6%
Gender				
Male	16	29.1%	37	29.6%
Female	39	70.9%	88	70.4%
Education level				
Below middle school	6	10.9%	78	62.4%
High school	7	12.7%	31	24.8%
Specialist	22	40.0%	11	8.8%
the University	13	23.6%	3	2.4%
master’s degree	5	9.1%	1	0.8%
PhD	2	3.6%	1	0.8%
Community service (participation) years				
Less than 1 year	6	10.9%	17	13.6%
Less than 1–3 years	13	23.6%	49	39.2%
Less than 3–6 years	23	41.8%	26	20.8%
Under 6–9 years	2	3.6%	8	6.4%
>9 years	11	20.0%	25	20.0%
Average number of days in the community a week				
1 day a week			8	6.4%
2 days a week			27	21.6%
3 days a week			11	8.8%
4 days a week			17	13.6%
More than 5 days a week			62	49.6%
Use body measurement equipment frequency				
Daily use			18	14.4%
Use 3 times a week			16	12.8%
Use once a week			81	64.8%
Use once a week			10	8.0%
Measured service experience				
Less than 6 months	4	7.3%		
6 months–1 year	27	49.1%		
1–3 years	20	36.4%		
3–5 years	3	5.5%		
5 years	1	1.8%		

**Table 3:** Reliability and validity analyses.

Research Participants	Dimensions		Items	Factor Loading	Cronbach’s α	Composite Reliability (CR)		Average Variance Extracted (AVE)
Volunteers	Information Quality		IQ1	0.879	0.840	0.904		0.758
			IQ2	0.888				
			IQ3	0.843				
	System Quality		SQ1	0.818	0.850	0.899		0.689
			SQ2	0.897				
			SQ3	0.812				
			SQ4	0.810				
	Use Satisfaction		US1	0.867	0.841	0.904		0.759
			US2	0.848				
			US3	0.898				
Participants of measurement service	Perceived Disease Threat		PDT1	0.854	0.891	0.924		0.753
			PDT2	0.879				
			PDT3	0.875				
			PDT4	0.864				
	Subjective Norms		SN1	0.796	0.634	0.841		0.727
			SN2	0.905				
	Behavioral Intention		BI1	0.935	0.947	0.966		0.904
			BI2	0.967				
			BI3	0.950				
	Active Aging	Psychology	AA1	0.947	0.932	0.949		0.825
			AA2	0929				
			AA3	0.857				
			AA4	0.897				
		Life	AA6	0.909	0.878	0.922		0.798
			AA7	0927				
			AA8	0.841				
		Social Participation	AA16	0.921	0.932	0.949		0.825
			AA17	0.825				
			AA18	0.930				
			AA19	0.793				
		Health	AA9	0.859	0.878	0.915		0.728
			AA10	0.855				
			AA11	0.866				
			AA12	0.834				
		Economy	AA13	0.881	0.790	0.875	0.701	
			AA14	0.899				
			AA15	0.720				

**Table 4:** Intercorrelations of the latent variables for Dimensions.

**Volunteers**								
Dimensions	IQ		SQ			US		
IQ	**0.870**							
SQ	0.812		**0.830**					
US	0.779		0.812			**0.871**		
**Participants of Measurement Service**								
Dimensions	AA Econ	AA Health	AA Phy	AA Social	AA life	BI	PDT	SN
AA_Econ	**0.837**							
AA_Health	0.765	**0.853**						
AA_Phy	0.767	0.814	**0.908**					
AA_Social	0.684	0.803	0.728	**0.869**				
AA_life	0.710	0.807	0.777	0.785	**0.893**			
BI	0.011	0.042	0.032	0.196	0.036	**0.951**		
PDT	−0.260	−0.295	−0.292	−0.232	−0.310	0.103	**0.868**	
SN	−0.146	−0.104	−0.100	−0.078	−0.097	0.652	0.347	**0.853**

**Table 6:** Basic information of Interviewee.

Encoding	Interview Code	Age	Education Level	Profession
Volunteer	A	42	University	Director General
	B	61	University	Volunteer
	C	67	University	Chairman

## Data Availability

The datasets used and/or analyzed during this study are available from the corresponding author on reasonable request.

## Appendix A

**Table A1:** Items of the questionnaire for volunteers.

System Quality [11,35]
I think this system is very stable.
I think this system is easy to use.
I think the operation of this system is easy to learn.
I think this system responds very quickly.
Information Quality [11,36]
This system can provide correct information without repeating operations.
The system information can be clearly displayed on the screen, making it easy for users to read.
The user interface of this system is well designed.
User Satisfaction [11,35]
You have a positive attitude or comment on this system function.
The function of this system meets my work requirements.
You are satisfied with this system.

**Table A2:** Items of the questionnaire for participants of the measurement service.

Perceived Disease Threat [37,38]	
I think I get sick easily.	
I think I may suffer from chronic diseases in the future.	
I feel that my health is worse than before.	
I may be forced to change my life due to chronic diseases in the future.	
Subjective Norms [39,40]	
People who influence my behavior would think that I should use the Baby machine.	
People who are important to me would think that I should use the Baby machine.	
Behavior Intention [41,42]	
I will use Baby machine on a regular basis in the future.	
I will frequently use Baby machine in the future.	
I will continue use Baby machine in the future.	
Active Aging [41,43,44]	
Psychological	Having satisfied one’s life goals.
	I like myself very much.
	Having positive attitude.
	I can appreciate the meaning of life.
	Acceptance of own mortality.
Life	Having pleasurable daily activities.
	I feel dependent on life.
	Being socially active.
Health	Pay attention to nutrition and health preservation.
	Having good health.
	Being able to take care of personal needs.
	Remaining in control of one’s life.
Economic	Having money to enjoy extras.
	Being financially secure.
	Can afford emergency medical expenses.
Social Participation	Interacting with others regularly.
	Being able to do things for others.
	Participating in various community activities makes my life more colorful.
	Having good friends.
